# Autophagy: More Than a Nonselective Pathway

**DOI:** 10.1155/2012/219625

**Published:** 2012-05-15

**Authors:** Fulvio Reggiori, Masaaki Komatsu, Kim Finley, Anne Simonsen

**Affiliations:** ^1^Department of Cell Biology and Institute of Biomembranes, University Medical Center Utrecht, 3584 CX Utrecht, The Netherlands; ^2^Protein Metabolism Project, Tokyo Metropolitan Institute of Medical Science, Tokyo 156-8506, Japan; ^3^BioScience Center, San Diego State University, San Diego, CA 921825, USA; ^4^Department of Biochemistry, Institute of Basic Medical Sciences, Faculty of Medicine, University of Oslo, 0317 Oslo, Norway

## Abstract

Autophagy is a catabolic pathway conserved among eukaryotes that allows cells to rapidly eliminate large unwanted structures such as aberrant protein aggregates, superfluous or damaged organelles, and invading pathogens. The hallmark of this transport pathway is the sequestration of the cargoes that have to be degraded in the lysosomes by double-membrane vesicles called autophagosomes. The key actors mediating the biogenesis of these carriers are the autophagy-related genes (*ATG*s). For a long time, it was assumed that autophagy is a bulk process. Recent studies, however, have highlighted the capacity of this pathway to exclusively eliminate specific structures and thus better fulfil the catabolic necessities of the cell. We are just starting to unveil the regulation and mechanism of these selective types of autophagy, but what it is already clearly emerging is that structures targeted to destruction are accurately enwrapped by autophagosomes through the action of specific receptors and adaptors. In this paper, we will briefly discuss the impact that the selective types of autophagy have had on our understanding of autophagy.

## 1. Introduction

Three different pathways can deliver cytoplasmic components into the lumen of the lysosome for degradation. They are commonly referred to as autophagy (cell “self-eating”) and include chaperone-mediated autophagy (CMA), microautophagy, and macroautophagy. CMA involves the direct translocation of specific proteins containing the KFERQ pentapeptide sequence across the lysosome membrane [1, 2]. Microautophagy, on the other hand, entails the invagination and pinching off of the lysosomal limiting membrane, which allows the sequestration and elimination of cytoplasmic components. The molecular mechanism underlying this pathway remains largely unknown. The only cellular function that so far has been indisputably assigned to microautophagy is the turnover of peroxisomes under specific conditions in fungi [3]. Recently, it has been reported the existence of a microautophagy-like process at the late endosomes, where proteins are selectively incorporated into the vesicles that bud inward at the limiting membrane of these organelles during the multivesicular bodies biogenesis [4]. In contrast to CMA and microautophagy, macroautophagy (hereafter referred to as autophagy) entails the formation of a new organelle, the autophagosome, which allows the delivery of a large number of different cargo molecules into the lysosome.

**Figure 1:**
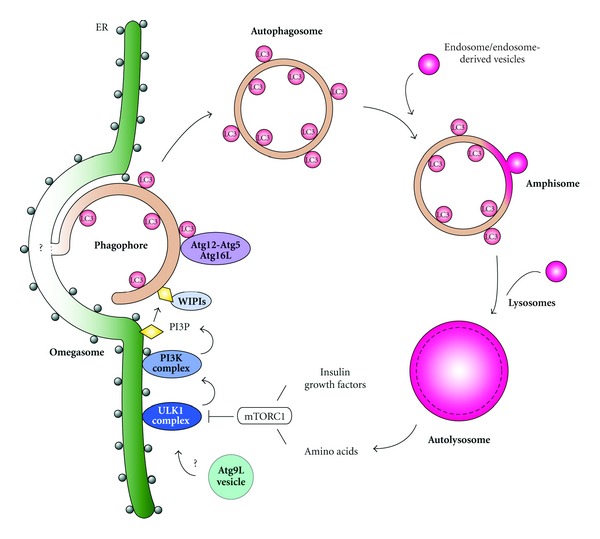
Multiple Atg proteins govern autophagosome formation. In response to inactivation of mTORC1 (but also other cellular and environmental cues), the ULK1 complex is activated and translocates in proximity of the endoplasmic reticulum (ER). Thereafter, the ULK1 complex regulates the class III PI3K complex. Atg9L, a multimembrane spanning protein, is also involved in an early stage of autophagosome formation by probably supplying part of the membranes necessary for the formation and/or expansion. Local formation of PI3P at sites called omegasomes promotes the formation of the phagophore, from which autophagosomes appear to be generated. The PI3P-binding WIPI proteins (yeast Atg18 homolog), as well as the Atg12-Atg5-Atg16L1 complex and the LC3-phosphatidylethanolamine (PE) conjugate play important roles in the elongation and closure of the isolation membrane. Finally, the complete autophagosome fuses with endosomes or endosome-derived vesicles forming the amphisome, which subsequently fuses with lysosomes to form autolysosomes. In the lysosomes, the cytoplasmic materials engulfed by the autophagosomes are degraded by resident hydrolases. The resulting amino acids and other basic cellular constituents are reused by the cell; when in high levels they also reactivate mTORC1 and then suppress autophagy.

Autophagy is a primordial and highly conserved intracellular process that occurs in most eukaryotic cells and participates in stress management. This pathway involves the *de novo* formation of vesicles called autophagosomes, which can engulf entire regions of the cytoplasm, individual organelles, protein aggregates, and invading pathogens (Figure 1). The autophagosomes fuse with endosomal compartments to form amphisomes prior to fusion with the lysosome, where their contents are degraded and the resulting metabolites are recycled back to the cytoplasm (Figure 1). Unique features of the pathway include the double-membrane structure of the autophagosomes, which were originally characterized over 50 years ago from detailed electron microscopy studies [5]. Starting in the 1990s yeast mutational studies began the genetic and molecular characterization of the key components required to initiate and build an autophagosome [6]. Subsequently, genetic and transgenic studies in plants, worms, fruit flies, mice, and humans have underscored the pathway's conservation and have begun to unveil the intricate vital role that autophagy plays in the physiology of cells and multicellular organisms.

For a long time, autophagy was considered a non-selective pathway induced as a survival mechanism in response to cellular stresses. Over the past several years, however, it has become increasingly evident that autophagy also is a highly selective process involved in clearance of excess or dysfunctional organelles, protein aggregates and intracellular pathogens. In this introductory piece, we will briefly discuss the molecular mechanisms of selective types of autophagy and their emerging importance as a quality control to maintain cellular and organismal health, aspects that will be presented in deep in the reviews of this special issue of the *International Journal of Cell Biology* and highlighted by the research papers.

## 2. The Mechanism of Autophagy

### 2.1. The Function of the Atg Proteins

Autophagosomes are formed by expansion and sealing of a small cistern known as the phagophore or isolation membrane (Figure 1). Once complete, they deliver their cargo into the hydrolytic lumen of lysosomes for degradation. A diverse set of components are involved in the biogenesis of autophagosomes, which primarily includes the proteins encoded by the autophagy-related genes (ATG). Most *ATG* genes have initially been identified and characterized in yeast. Subsequent studies in higher eukaryotes have revealed that these key factors are highly conserved. To date, 36 Atg proteins have been identified and 16 are part of the core Atg machinery essential for all autophagy-related pathways [7]. Upon autophagy induction, these proteins associate following a hierarchical order [8, 9] to first mediate the formation of the phagophore and then to expand it into an autophagosome [10, 11]. While their molecular functions and their precise contribution during the biogenesis of double-membrane vesicles remain largely unknown, they have been classified in 4 functional groups of genes: (1) the Atg1/ULK complex, (2) the phosphatidylinositol 3-kinase (PI3K) complex, (3) the Atg9 trafficking system, and (4) the two parallel ubiquitin-like conjugation systems (Figure 1).

The Atg1/ULK complex consists of Atg1, Atg13, and Atg17 in yeast, and ULK1/2, Atg13, FIP200 and Atg101 in mammals [12–15]. This complex is central in mediating the induction of autophagosome biogenesis and as a result it is the terminal target of various signaling cascades regulating autophagy, such as the TOR, insulin, PKA, and AMPK pathways [16] (Figure 1). Increased activity of the Atg1/ULK kinase is the primary event that determines the acute induction and upregulation of autophagy. It is important to note that ULK1 is part of a protein family and two other members, ULK2 and ULK3, have been shown play a role in autophagy induction as well [14, 17]. The expansion of this gene family may reflect the complex regulation and requirements of the pathway in multicellular long-lived organisms. Stimulation of the ULK kinases is achieved through an intricate network of phosphorylation and dephosphorylation modifications of the various subunits of the Atg1/ULK complex. For example, Atg13 is directly phosphorylated by TOR and the phosphorylation state of Atg13 modulates its binding to Atg1 and Atg17. Inactivation of TOR leads to a rapid dephosphorylation of Atg13, which increases Atg1–Atg13–Atg17 complex formation, stimulates the Atg1 kinase activity and induces autophagy [18, 19]. The mAtg13 is also essential for autophagy, but seems to directly interact with ULK1, ULK2 and FIP200 independently of its phosphorylation state [13, 14]. In addition, there are several phosphorylation events within this complex as well, including phosphorylation of mAtg13 by ULK1, ULK2, and TOR; phosphorylation of FIP200 by ULK1 and ULK2; phosphorylation of ULK1 and ULK2 by TOR [13, 14]. Additional studies are required to fully characterize the functional significance of these posttranslational modifications.

Autophagy is also regulated by the activity of PI3K complexes. Yeast contains a single PI3K, Vps34, which is present in two different tetrameric complexes that share 3 common subunits, Vps34, Vps15, and Atg6 [20]. Complex I is required for the induction of autophagy and through its fourth component, Atg14, associates to the autophagosomal membranes where the lipid kinase activity of Vps34 is essential for generating the phosphatidylinositol-3-phosphate (PI3P) that permits the recruitment of other Atg proteins [9, 21] (Figure 1). Complex II contains Vps38 as the fourth subunit and it is involved in endosomal trafficking and vacuole biogenesis [20]. There are three types of PI3K in mammals: class I, II, and III. The functions of class II PI3K remains largely unknown, but both classes I and III PI3Ks are involved in autophagy. While class I PI3K is principally implicated in the modulation of signalling cascades, class III PI3K complexes regulate organelle biogenesis and, like yeast, contain three common components: hVps34, p150 (Vps15 ortholog), and Beclin 1 (Atg6 ortholog). The counterparts of Atg14 and Vps38 are called Atg14L/Barkor and UVRAG, respectively [22–24]. The Atg14L-containing complex plays a central role in autophagy and functions very similarly as the yeast complex I by directing the class III PI3K complex I to the phagophore to produce PI3P and initiate the recruitment of the Atg machinery (Figure 1). Atg14L is thought to be present on the ER irrespective of autophagy induction [25]. Upon starvation, Atg14L localizes to autophagosomal membranes [8]. Importantly, depletion of Atg14L reduces PI3P production, impairs the formation of autophagosomal precursor structures, and inhibits autophagy [8, 24, 26, 27]. The UVRAG-containing class III PI3K complex also regulates autophagy but it appears to act at the intersection between autophagy and the endosomal transport pathways. UVRAG initially associates with the BAR-domain protein Bif-1, which may regulate mAtg9 trafficking from the trans-Golgi network (TGN) [28, 29]. UVRAG then interacts with the class C Vps/HOPS protein complex, promoting the fusion of autophagosomes with late endosomes and/or lysosomes [30]. Finally, the UVRAG-containing class III protein complex binds to Rubicon, a late endosomal and lysosomal protein that suppresses autophagosome maturation by reducing hVps34 activity [26, 31]. Importantly, both the Atg14L- and UVRAG-containing complexes interact through Beclin 1 with Ambra1, which in turn tethers these protein complexes to the cytoskeleton via an interaction with dynein [32, 33]. Following the induction of autophagy, ULK1 phosphorylates Ambra1 thus releasing the class III PI3K complexes from dynein and their subsequent relocalization triggers autophagosome formation. Therefore, Ambra1 constitutes a direct regulatory link between the Atg1/ULK1 and the PI3K complexes [32].

Together with the Atg1/ULK and the PI3K complexes, Atg9 is one of the first factors localizing to the preautophagosomal structure or phagophore assembly site (PAS), the structure believed to be the precursor of the phagophore [9, 34] (Figure 1). Atg9 is the only conserved transmembrane protein that is essential for autophagy. It is distributed to the PAS and multiple additional cytoplasmic tubulovesicular compartments derived from the Golgi [35–37]. Atg9 cycles between these two locations and consequently it is thought to serve as a membrane carrier providing the lipid building blocks for the expanding phagophore [37]. One of the established functions of Atg9 is that it leads to the formation of the yeast PAS when at least one of the cytoplasmic tubulovesicular compartments translocates near the vacuole [34]. Atg9 is also essential to recruit the PI3K Complex I to the PAS [9]. Retrieval transport of yeast Atg9 from the PAS and/or complete autophagosome is mediated by the Atg2-Atg18 complex [38] and appears to be regulated by the Atg1/ULK and PI3K complexes [37]. Mammalian Atg9 (mAtg9) has similar characteristics to its yeast counterpart. mAtg9 localizes to the TGN and late endosomes and redistributes to autophagosomal structures upon the induction of autophagy (Figure 1) [39], further promoting pathway activity [29, 40–42]. As in yeast, cycling of mAtg9 between locations also requires the Atg1/ULK complex and kinase activity hVps34 [39, 43].

The core Atg machinery also entails two ubiquitin-like proteins, Atg12 and Atg8/microtubule-associated protein 1 (MAP1)-light chain 3 (LC3), and their respective, partially overlapping, conjugation systems [44–46] (Figure 1). Atg12 is conjugated to Atg5 through the activity of the Atg7 (E1-like) and the Atg10 (E2-like) enzymes. The Atg12–Atg5 conjugate then interacts with Atg16, which oligomerizes to form a large multimeric complex. Atg8/LC3 is cleaved at its C terminus by the Atg4 protease to generate the cytosolic LC3-I with a C-terminal glycine residue, which is then conjugated to phosphatidylethanolamine (PE) in a reaction that requires Atg7 and the E2-like enzyme Atg3. This lipidated form of LC3 (LC3-II) is attached to both faces of the phagophore membrane. Once the autophagosome is completed, Atg4 removes LC3-II from the outer autophagosome surface. These two ubiquitination-like systems appear to be closely interconnected. On one hand, the multimeric Atg12-Atg5-Atg16 complex localizes to the phagophore and acts as an E3-like enzyme, determining the site of Atg8/LC3 lipidation [47, 48]. On the other hand, the Atg8/LC3 conjugation machinery seems to be essential for the optimal functioning of the Atg12 conjugation system. In Atg3-deficient mice, Atg12-Atg5 conjugation is markedly reduced, and normal dissociation of the Atg12-Atg5-Atg16 complex from the phagophore is delayed [49]. Some evidences suggest that these two conjugation systems also function together during the expansion and closure of the phagophore. For example, overexpression of an inactive mutant of Atg4 inhibits the lipidation of LC3 and leads to the accumulation of a number of nearly complete autophagosomes [47]. While controversial [50], it has been postulated that Atg8/LC3 also possesses fusogenic properties, thus mediating the assembly of the autophagic membrane [51, 52].

It has to be noted that mammals possess at least 7 genes coding for LC3/Atg8 proteins that can be grouped into three subfamilies: (1) the LC3 subfamily containing LC3A, LC3B, LC3B2 and LC3C; (2) the gammaaminobutyrate receptor-associated protein (GABARAP) subfamily comprising GABARAP and GABARAPL1 (also called GEC-1); (3) the Golgi-associated ATPase enhancer of 16 kDa (GATE-16) protein (also called GABARAP-L2/GEF2) [53]. Although *in vivo* studies show that they are all conjugated to PE, they appear to have evolved complex nonredundant functions [54].

### 2.2. The Autophagosomal Membranes

The origin of the membranes composing autophagosomes is a long-standing mystery in the field of autophagy. A major difficulty in addressing this question has been that phagophores as well as autophagosomes do not contain marker proteins of other subcellular compartments [55, 56]. A series of new studies has implicated several cellular organelles as the possible source for the autophagosomal lipid bilayers. The plasma membrane and elements of the trafficking machinery to the cell surface have been linked to the formation of an early autophagosomal intermediate, perhaps the phagophore [57–61]. It is possible that early endosomal- and/or Golgi-derived membranes are also key factors in the initial steps of autophagy [34, 36, 39]. The Golgi, moreover, appears also important for autophagy by supplying at least in part the extra lipids required for the phagophore expansion [29, 62–65]. The endoplasmic reticulum (ER) is also central in this latter event. While the relevance of the ER in autophagosome biogenesis was already pointed out a long time ago [5, 55, 66, 67], recently two electron tomography studies have demonstrated the existence of a physical connection between the ER and the forming autophagosomes [68, 69]. These analyses have revealed that the ER is connected to the outer as well as the inner membrane of the phagophore through points of contact, supporting the notion that lipids could be supplied via direct transfer at the sites of membrane contact. In line with this view, it has been found that Atg14L is associated to the ER and PI3P is generated on specific subdomains of this organelle from where autophagosomes emerge under autophagy-inducing conditions [25, 70] (Figure 1). It has also been proposed that the outer membrane of the mitochondria is the main source of the autophagosomal lipid bilayers, but while the experimental evidences appear to show that mitochondria are essential for the phagophore expansion, it remains unclear whether these organelles play a key role in the phagophore biogenesis [71]. The discrepancy between the conclusions of the various studies has not allowed yet drawing a model about the membrane dynamics during autophagosome biogenesis. The different results could be due to the different experimental conditions and model systems used by the various laboratories. Alternatively, the lipids forming the autophagosomes could have different sources depending on the cell and the conditions inducing autophagy [72, 73]. A third possibility is that the source of phagophore membrane could depend on the nature of the double-membrane vesicle cargo. Additional investigations are required to shed light on these issues.

### 2.3. Pharmacological Manipulation of Autophagy

Despite the potential of curing, quite a substantial range of specific pathological conditions by inducting autophagy, there are currently no small molecules that allow to exclusively stimulate this pathway [74]. Nevertheless, there is a variety of chemicals that by acting on signaling cascades that also regulate autophagy permit to trigger this degradative process. These agents fall into two distinct categories based on the mechanism of action; whether they work through an mTOR-dependent (Rapamycin or Torin) or mTOR-independent pathway (e.g., lithium or resveratrol) [74]. In addition to these compounds, there are biological molecules such as interferon *γ* (IFN*γ*) and vitamin D that can be used to stimulate autophagy especially in experimental setups [75, 76].

Inhibition of autophagy can also be beneficial in specific diseases but as for the inducers there are no compounds that exclusively block this pathway without affecting other cellular processes. The small molecules inhibiting autophagy include wortmannin and 3-methyladenine, which hamper the activity of the PI3K; Bafilomycin A and chloroquine, which impair the degradative activity of lysosomes [77]. They are currently solely used in the basic research on autophagy.

## 3. Selective Types of Autophagy

### 3.1. The Molecular Machinery of Selective Autophagy

It is becoming increasingly evident that autophagy is a highly selective quality control mechanism whose basal levels are important to maintain cellular homeostasis (see below). A number of organelles have been found to be selectively turned over by autophagy and cargo-specific names have been given to distinguish the various selective pathways, including the ER (reticulophagy or ERphagy), peroxisomes (pexophagy), mitochondria (mitophagy), lipid droplets (lipophagy), secretory granules (zymophagy), and even parts of the nucleus (nucleophagy). Moreover, pathogens (xenophagy), ribosomes (ribophagy), and aggregate-prone proteins (aggrephagy) are specifically targeted for degradation by autophagy [78].

Selective types of autophagy perform a cellular quality control function and therefore they must be able to distinguish their substrates, such as protein aggregates or dysfunctional mitochondria, from their functional counterparts. The molecular mechanisms underlying cargo selection and regulation of selective types of autophagy are still largely unknown. This has been an area of intense research during the last years and our understanding of the various selective types of autophagy is starting to unravel. A recent genome-wide small interfering RNA screen aimed at identifying mammalian genes required for selective autophagy found 141 candidate genes to be required for viral autophagy and 96 of those genes were also required for Parkin-mediated mitophagy [79].

In general, these pathways appear to rely upon specific cargo-recognizing autophagy receptors, which connect the cargo to the autophagic membranes. The autophagy receptors might also interact with specificity adaptors, which function as scaffolding proteins that bring the cargo-receptor complex in contact with the core Atg machinery to allow the specific sequestration of the substrate. The selective types of autophagy appear to rely on the same molecular core machinery as non-selective (starvation-induced) bulk autophagy. In contrast, the autophagy receptors and specificity adapters do not seem to be required for nonselective autophagy.

Autophagy receptors are defined as proteins being able to interact directly with both the autophagosome cargo and the Atg8/LC3 family members through a specific (WxxL) sequence [80], commonly referred to as the LC3-interacting region (LIR) motif [81] or the LC3 recognition sequences (LRS) [82]. Based on comparison of LIR domains from more than 20 autophagy receptors it was found that the LIR consensus motif is an eight amino acids long sequence that can be written D/E-D/E-D/E-W/F/Y-X-X-L/I/V. Although not an absolute requirement, usually there is at least one acidic residue upstream of the W-site. The terminal L-site is occupied by a hydrophobic residue, either L, I, or V [83]. The LIR motifs of several autophagy receptors have been found to interact both with LC3 and GABARAP family members *in vitro*, but whether this reflects a physiological interaction remains to be clarified in most cases. It should be pointed out that not all LIR-containing proteins are autophagy cargo receptors. Some LIR-containing proteins, like Atg3 and Atg4B, are recruited to autophagic membranes to perform their function in autophagosome formation [84, 85], whereas others like FYVE and coiled-coil domain-containing protein 1 (FYCO1) interact with LC3 to facilitate autophagosome transport and maturation [86]. Others might use an LIR motif to become degraded, like Dishevelled, an adaptor protein in the Wnt signalling pathway [87]. The adaptor proteins are less well-described, but seem to interact with autophagy receptors and work as scaffold proteins recruiting and assembling the Atg machinery required to generate autophagosomes around the cargo targeted to degradation. Examples of autophagy adaptors are Atg11 and ALFY [88, 89].

**Figure 2:**
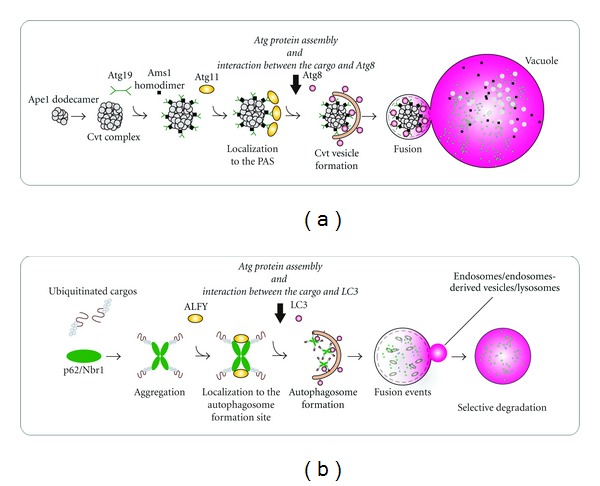
Representative selective autophagy. (a) The cytoplasm-to-vacuole targeting (Cvt) pathway. Ape1 is synthesized as a cytoplasmic precursor protein with a propeptide and rapidly oligomerizes into dodecamers that subsequently associate with each other to form a higher order complex. The autophagy receptor Atg19 directly binds to the complex and mediates the recruitment of another Cvt pathway cargo, Ams1, leading to the formation of the so-called Cvt complex. Atg19 also interacts with the autophagy adaptor Atg11 and this protein allows the transport of the Cvt complex to the site where the double-membrane vesicle will be generated. At this location, Atg11 tethers the Atg proteins essential for the Cvt vesicle formation and the direct binding of Atg19 to Atg8 permits the exclusive sequestration of the Cvt complex into the vesicle. (b) A model for p62 and NBR1 as autophagy receptors for ubiquitinated cargos. p62 and NBR1 bind with ubiquitinated cargos via their ubiquitin-associated (UBA) domain and this interaction triggers the aggregate formation through the oligomerization of p62 via its Phox and Bem1p (PB1) domain. Furthermore, p62 interacts with both autophagy-linked FYVE protein (ALFY), which serves to recruit Atg5 and to bind PI3P, and directly with LC3. This latter event appears to organize and activate the Atg machinery in close proximity of the ubiquitinated cargos, which allows to selectively sequester them in the autophagosomes in analogous to the Cvt pathway.

The list of specific autophagy receptors is rapidly growing and the role of several of them in different types of selective autophagy will be described in detail in the reviews of this special issue. Here we will briefly discuss the best studied form of selective autophagy, the yeast cytosol to vacuole targeting (Cvt) pathway, as well as the best studied mammalian autophagy receptor, p62/sequestosome 1 (SQSTM1) (Figure 2).

The Cvt pathway is a biosynthetic process mediating the transport of the three vacuolar hydrolases, aminopeptidase 1 (Ape1), aminopeptidase 4 (Ape4) and *α*-mannosidase (Ams1), and the Ty1 transposome into the vacuole [90, 91]. Ape1 is synthesized as a cytosolic precursor (prApe1), which multimerizes into the higher order Ape1 oligomer, to which Ape4, Ams1, and Ty1 associate to form the so-called Cvt complex, prior to being sequestered into a small autophagosome-like Cvt vesicle. Sequestration of the Cvt complex into Cvt vesicles is a multistep process, which requires the autophagy receptor Atg19, which facilitates binding to Atg8 at the PAS, as well as the adaptor protein Atg11 (Figure 2(a)) [92]. Atg11 acts as a scaffold protein by directing the Cvt complex and Atg9 reservoirs translocation to the PAS in an actin-dependent way and then recruiting the Atg1/ULK complex [40, 93]. The PI3P-binding proteins Atg20, Atg21, and Atg24 are also required for the Cvt pathway [94, 95], but their precise function remains to be elucidated. Interestingly, Atg11 overexpression was found to recruit more Atg8 and Atg9 to the PAS resulting in more Cvt vesicles. This observation indicates that Atg11 levels could regulate the rate of selective autophagy, and maybe also the size of the cargo-containing autophagosomes in yeast [90, 96]. Indeed, a series of studies has revealed that Atg11 is also involved in other types of selective autophagy such as mitophagy and pexophagy. However, the autophagy receptors involved in the different Atg11-dependent types of selective autophagy are different as Atg32 is required for mitophagy [97, 98], whereas Atg30 is essential for pexophagy [99]. Like Atg19, these two proteins have an Atg8-binding LIR motif and directly interact with Atg11. Mammalian cells appear to not possess an Atg11 homologue, and further studies are necessary to delineate the molecular machinery involved in sequestration and targeting of different cargoes for degradation by autophagy in higher eukaryotes.

The mechanism of the Cvt pathway is reminiscent of the selective form of mammalian autophagy called aggrephagy, which involves degradation of misfolded and unwanted proteins by packing them into ubiquitinated aggregates. In both cases aggregation of the substrate (prApe1 or misfolded proteins) is required prior to sequestration into Cvt vesicles or autophagosomes, respectively [100–102]. Similar to Cvt vesicles, aggregate-containing autophagosomes appear to be largely devoid of cytosolic components suggesting that the vesicle membrane expands tightly around its cargo [88]. Aggrephagy also depends on proteins with exclusive functions in substrate selection and targeting [81, 88, 100, 103]. The autophagy receptors p62 and neighbour of BRCA1 gene (NBR1) bind both ubiquitinated protein aggregates through an ubiquitin-associated (UBA) domain and to LC3 via their LIR motifs and, thereby, promote the specific autophagic degradation of ubiquitinated proteins (Figure 2(b)) [81, 82, 100, 103, 104]. NBR1 and p62 also contain an N-terminal Phox and Bem1p (PB1) domain through which they can oligomerize, or interact with other PB1-containing binding partners [83]. In addition to being a cargo receptor for protein aggregates, p62 has been implicated in autophagic degradation of other ubiquitinated substrates such as intracellular bacteria [105], viral capsid proteins [106], the midbody remnant formed after cytokinesis [107], peroxisomes [108, 109], damaged mitochondria [110, 111], and bacteriocidal precursor proteins [112]. The PB1 domain was recently found to be required for p62 to localize to the autophagosome formation site adjacent to the ER [113], suggesting that it could target ubiquitinated cargo to the site of autophagosome formation or alternatively promote the assembly of the Atg machinery at this location.

The large scaffolding protein autophagy-linked FYVE (ALFY) appears to have a similar function as the specificity adaptor Atg11. ALFY is recruited to aggregate-prone proteins through its interaction with p62 [101] and through a direct interaction with Atg5 and PI3P it serves to recruit the core Atg machinery and allow formation of autophagic membranes around the protein aggregate [88] (Figure 2(b)). Interestingly, ALFY is recruited from the nucleus to cytoplasmic ubiquitin-positive structures upon cell stress suggesting that it might regulate the level of aggrephagy [114]. In line with this, it was found that overexpression of ALFY in mouse and fly models of Huntington's disease reduced the number of protein inclusions [88]. It will be interesting to determine whether ALFY, as p62, is involved in other selective types of autophagy such as the one eliminating midbody ring structures or mitochondria.

### 3.2. Regulation of Selective Autophagy

It is well known that posttranslational modifications like phosphorylation and ubiquitination are involved in the regulation of the activity of proteins involved in autophagy and degradation of autophagic cargo proteins, respectively. However, little is known about how these modifications may regulate selective autophagy. The fact that the core Atg machinery is required for both nonselective and selective types of autophagy gives raise to the question of whether these two types of autophagy may compete for the same molecular machinery. Such a competition could be detrimental for the cells undergoing starvation and to avoid this, there might be a tight regulation of the expression level and/or activity of the proteins specifically involved the selective autophagy. It has recently been proposed that phosphorylation of autophagy receptors might be a general mechanism for the regulation of selective autophagy. Dikic and coworkers noted that several autophagy receptors contain conserved serine residues adjacent to their LIR motifs and indeed, the TANK binding kinase 1 (TBK1) was found to phosphorylate a serine residue close to the LIR motif of the autophagy receptor optineurin. This modification enhances the LC3 binding affinity of optineurin and promotes selective autophagy of ubiquitinated cytosolic *Salmonella enterica *[115]. In yeast, phosphorylation of Atg32, the autophagy receptor for mitophagy, by mitogen-activated protein kinases was found to be required for mitophagy [116, 117].

The Atg8/LC3 proteins themselves have also been found to become phosphorylated and recent works have identified specific phosphorylation sites for protein kinase A (PKA) [118] and protein kinase C (PKC) [119] in the N-terminal region of LC3. Interestingly, the N-terminal of LC3 is involved in the binding of LC3 to LIR-containing proteins [120]. It is therefore tempting to speculate that phosphorylation of the PKA and PKC sites might facilitate or prevent the interaction of LC3 with LIR-containing proteins such as p62. It has been found that phosphorylation of the PKA site, which is conserved in all mammalian LC3 isoforms, but not in GABARAP, inhibits recruitment of LC3 into autophagosomes [118].

The role of ubiquitin in autophagy has so far been ascribed as a signal for cargo degradation. Ubiquitination of aggregate prone proteins, as well as bacteria and mitochondria, has been found to serve as a signal for recognition by autophagy receptors like p62 and NBR1, which are themselves also degraded together with the cargo that they associate with [83]. The *in vivo* specificity of p62 and NBR1 toward ubiquitin signals remains to be established under the different physiological conditions. Interestingly, it was recently found that casein kinase 2- (CK2-) mediated phosphorylation of the p62 UBA domain increases the binding affinity of this motif for polyubiquitin chains leading to more efficient targeting of polyubiquitinated proteins to autophagy [121]. CK2 overexpression or phosphatase inhibition reduced the formation of aggregates containing the polyglutamine-expanded huntingtin exon1 fragments in a p62-dependent manner. The E3 ligases involved in ubiquitination of different autophagic cargo largely remains to be identified. However, it is known that the E3 ligases Parkin and RNF185 both regulate mitophagy [122, 123]. SMURF1 (SMAD-specific E3 ubiquitin protein ligase 1) was recently also implicated in mitophagy, as well as in autophagic targeting of viral particles [79]. Interestingly, the role of SMURF1 in selective autophagy seems to be independent of its E3 ligase activity, but it rather depends on its membrane-targeting C2 domain, although the exact mechanism involved remains to be elucidated. It is also not clear whether ubiquitination could serve as a signal to regulate the activity or binding selectivity of proteins directly involved in autophagy, and whether this in some way could regulate selective autophagy. The role of ubiquitin-like proteins as SUMO and Nedd in autophagy is also unexplored.

Acetylation is another posttranslational modification that only recently has been implicated in selective autophagy. The histone de-acetylase 6 (HDAC6), initially found to mediate transport of misfolded proteins to the aggresome [124], was lately implicated in maturation of ubiquitin-positive autophagosomes [125]. The fact that HDAC6 overproduction in fly eyes expressing expanded polyQ proteins is neuroprotective further indicates that HDAC6 activity stimulates aggrephagy [126]. Furthermore, the acetylation of an aggrephagy cargo protein, muntant huntingtin, the protein causing Huntington's disease, is important for its degradation by autophagy [127]. HDAC6 has been also implicated in Parkin-mediated clearance of damaged mitochondria [128]. The acetyl transferase(s) involved in these forms of selective autophagy is currently unknown, but understanding the role of acetylation in relation to various aspects of autophagy is an emerging field and it will very likely provide more mechanistic insights into these pathways.

## 4. Pathophysiological Relevance of Selective Types of Autophagy

**Figure 3:**
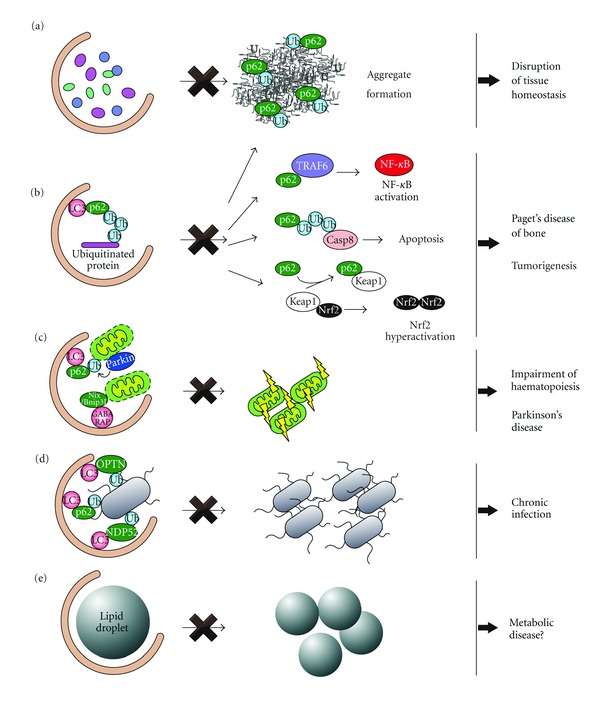
Pathophysiological relevance of selective autophagy. (a, b) Selective types of autophagy operates constitutively at low levels even under nutrient-rich conditions and mediates turnover of selected cytoplasmic materials through the action of autophagy receptors such as p62 and NBR1. These proteins mediate the elimination of ubiquitinated structures, including protein aggregates (a) and defects in these pathways lead to the disruption of tissue homeostasis, resulting in life-threatening diseases. Defective autophagy is usually accompanied by extensive accumulation of p62-containing aggregates, which enhances its function as a scaffold protein in several signaling cascades such as NF-*κ*B signaling, apoptosis, and Nrf2 activation (b). Such abnormalities might be involved in tumorigenesis and Paget's disease of bone. (c) During erythroid differentiation, Nix/Bnip3L relocalization to mitochondria leads to their depolarization, which triggers mitophagy. Loss of *Nix/Bnip3L* causes an arrest in the erythroid maturation arrest, leading to severe anaemia. In response to loss of the mitochondrial membrane potential, Parkin translocates onto the damaged mitochondria in a PINK1-dependent manner, and ubiquitinated proteins present on the outer mitochondrial membrane, which induces mitophagy. Parkinson's disease-related mutations in the *Parkin* and *PINK1* genes provoke a defect in mitophagy, suggesting this selective type of autophagy has a role in preventing the pathogenesis of the Parkinson's disease. (d) Specific bacteria invading the cytosol get ubiquitinated and are recognized by autophagy receptors such as p62, NDP52, and optineurin (OPTN). This allows the specific sequestration of the microbes into autophagosomes and their delivery into the lysosomes for degradation. (e) The lipid droplets are probably degraded by autophagy selectively. This selective type of autophagy, lipophagy, supplies free-fatty acids utilized to generate energy through the *β*-oxidation. Impairments in lipophagy are known to cause accumulation of lipid droplets in hepatocytes and reduced production of AgRP in neurons.

Basal autophagy acts as the quality control pathway for cytoplasmic components and it is crucial to maintain the homeostasis of various postmitotic cells [129]. While this quality control could be partially achieved by nonselective autophagy, growing lines of evidence have demonstrated that specific proteins, organelles, and invading bacteria are specifically degraded by autophagy (Figure 3).

### 4.1. Tissue Homeostasis

Mice deficient in autophagy die either *in utero* (e.g.,* Beclin 1* and *Fip200 *knockout mice) [130–132] or within 24 hours after birth due, at least in part, to a deficiency in the mobilization of amino acids from various tissues (e.g.,* Atg3, Atg5*, *Atg7*, *Atg9,* and *Atg16L* knockout mice) [49, 133–136]. As a result, to investigate the physiological roles of autophagy, conditional knockout mice for *Atg5*, *Atg7,* or *FIP200* and various tissue-specific *Atg* knockout mice have been established and analyzed [133, 137, 138]. For example, the liver-specific *Atg7-*deficient mouse displayed severe hepatomegaly accompanied by hepatocyte hypertrophy, resulting in severe liver injuries [133]. Mice lacking *Atg5*, *Atg7,* or *FIP200* in the central nervous system exhibited behavioral deficits, such as abnormal limb-clasping reflexes and reduction of coordinated movement as well as massive neuronal loss in the cerebral and cerebellar cortices [137–139]. Loss of *Atg5* in cardiac muscle caused cardiac hypertrophy, left ventricular dilatation, and systolic dysfunction [140]. Skeletal muscle-specific *Atg5* or *Atg7* knockout mice showed age-dependent muscle atrophy [141, 142]. Pancreatic *β* cell-specific *Atg7* knockout animals exhibited degeneration of islets and impaired glucose tolerance with reduced insulin secretion [143, 144]. Podocyte-specific deletion of *Atg5* caused glomerulosclerosis in aging mice and these animals displayed increased susceptibility to proteinuric diseases caused by puromycin aminonucleoside and adriamycin [145]. Proximal tubule-specific *Atg5 *knockout mice were susceptible to ischemia-reperfusion injury [146]. Finally, deletion of *Atg7* in bronchial epithelial cells resulted in hyperresponsiveness to cholinergic stimuli [147]. All together, these results undoubtedly indicate that basal autophagy prevents numerous life-threatening diseases.

How does impairment of autophagy lead to diseases? Ultrastructural analyses of the mutant mice revealed a marked accumulation of swollen and deformed mitochondria in the mutant hepatocytes [133], pancreatic *β* cells [143, 144], cardiac and skeletal myocytes [140, 141] and neurons [138], but also the appearance of concentric membranous structures consisting of ER or sarcoplasmic reticulum in hepatocytes [133], neuronal axons [137, 139] and skeletal myocytes [141], as well as an increased number of peroxisomes and lipid droplets in hepatocytes [133, 148]. In addition to the accumulation of aberrant organelles, histological analyses of tissues with defective autophagy showed the amassment of polyubiquitylated proteins in almost all tissues (although the level varied from one region to another) forming inclusion bodies whose size and number increased with aging [149]. Consequently, basal autophagy also acts as the quality control machinery for cytoplasmic organelles (Figure 3(a)). Although this could be partially achieved by bulk autophagy, these observations point to the existence of selective types of autophagy, a notion that is now supported by experimental data.

### 4.2. Implications of Selective Degradation of p62 by Autophagy

p62/SQSTM1 is the best-characterized disease-related autophagy receptor and a ubiquitously expressed cellular protein conserved among metazoan but not in plants and fungi [83]. Besides a role of p62 as the receptor, this protein itself is specific substrate for autophagy. Suppression of autophagy is usually accompanied by an accumulation of p62 mostly in large aggregates also positive for ubiquitin (Figure 3(a)) [104, 150]. Ubiquitin and p62-positive inclusion bodies have been detected in numerous neurodegenerative diseases (i.e., Alzheimer's disease, Parkinson's disease, and amyotrophic lateral sclerosis), liver disorders (i.e., alcoholic hepatitis and steatohepatitis), and cancers (i.e., malignant glioma and hepatocellular carcinoma) [151]. Very interestingly, the p62-positive aggregates observed in hepatocytes and neurons of liver- and brain-specific *Atg7* deficient mice, respectively, as well as in human hepatocellular carcinoma cells, are completely dispersed by the additional loss of p62 strongly implicating involvement of p62 in the formation of disease-related inclusion bodies [104, 152].

Through its self-oligomerization, p62 is involved in several signal transduction pathways. For example, this protein functions as a signaling hub that may determine whether cells survive by activating the TRAF6–NF-*κ*B pathway, or die by facilitating the aggregation of caspase 8 and the downstream effector caspases [153, 154]. On the other hand, p62 interacts with the Nrf2-binding site on Keap1, a component of the Cullin 3-type ubiquitin ligase for Nrf2, resulting in stabilization of Nrf2 and transcriptional activation of Nrf2 target genes including a battery of antioxidant proteins [155–159]. It is thus plausible that excess accumulation or mutation of p62 leads to hyperactivation of these signaling pathways, resulting in a disease onset (Figure 3(b)).

Paget's disease of bone is a chronic and metabolic bone disorder that is characterized by an increased bone turnover within discrete lesions throughout the skeleton. Mutations in the *p62* gene, in particular in its UBA domain, can cause this illness [160]. A proposed model explaining how p62 mutations lead to the Paget's disease of bone is the following: mutations of the UBA domain cause an impairment in the interaction between p62 and ubiquitinated TRAF6 and/or CYLD, an enzyme deubiquitinating TRAF6, which in turn enhances the activation of the NF-*κ*B signaling pathway and the resulting increased osteoclastogenesis (Figure 3(b)) [160]. If proven, this molecular scenario could open the possibility of using autophagy enhancers as a therapy to cure Paget's disease of bone.

It is established that autophagy has a tumor-suppressor role and several autophagy gene products including Beclin1 and UVRAG are known to function as tumor suppressor proteins [161]. The tumor-suppressor role of autophagy appears to be important particularly in the liver. Spontaneous tumorigenesis is observed in the livers of mice with either a systemic mosaic deletion of *Atg5* or a hepatocyte-specific *Atg7* deletion [152, 162]. Importantly, no tumors are formed in other organs in *Atg5* mosaically deleted mice. Enlarged mitochondria, whose functions are at least partially impaired, accumulate in *Atg5-* or *Atg7-*deficient hepatocytes [152, 162]. This observation is in line with the previous data obtained in iBMK cell lines showing that both the oxidative stress and genomic damage responses are activated by loss of autophagy [163, 164]. Again, it is clear that accumulation of p62, at least partially, contributes to tumor growth because the size of the *Atg7^−/−^* liver tumors is reduced by the additional deletion of *p62 *[162], which may cause a dysregulation of NF-*κ*B signaling [165] and/or a persistent activation of Nrf2 [166].

### 4.3. Selective Degradation of Ubiquitinated Proteins

Almost all tissues with defective autophagy are usually displaying an accumulation of polyubiquitinated proteins [149]. Loss of autophagy is considered to lead to a delay in the global turnover of cytoplasmic components [137] and/or to an impaired degradation of substrates destined for the proteasome [167]. Both observations could partially explain the accumulation of misfolded and/or unfolded proteins that is followed by the formation of inclusion bodies.

As discussed above, p62 and NBR1 act as autophagy receptors for ubiquitinated cargos such as protein aggregates, mitochondria, midbody rings, bacteria, ribosomal proteins and virus capsids [83, 168] (Figure 3). Although these studies suggest the role of p62 as an ubiquitin receptor, it remains to be established whether soluble ubiquitinated proteins are also degraded one-by-one by p62 and possibly NBR1. A mass spectrometric analysis has clearly demonstrated the accumulation of all detectable topologies of ubiquitin chain in *Atg* deficient livers and brains, indicating that specific polyubiquitin chain linkage is not the decisive signal for autophagic degradation [169]. Because the increase in ubiquitin conjugates in the *Atg7* deficient liver and brain is completely suppressed by additional knockout of either *p62* or *Nrf2* [169], accumulation of ubiquitinated proteins in tissues defective in autophagy might be attributed to p62-mediated activation of Nrf2, resulting in global transcriptional changes to ubiquitin-associated genes. Further studies are needed to precisely elucidate the degradation mechanism of soluble ubiquitinated proteins by autophagy.

### 4.4. Mitophagy

Concomitant with the energy production through oxidative phosphorylation, mitochondria also generate reactive oxygen species (ROS), which cause damage through the oxidation of proteins, lipids and DNA often inducing cell death. Therefore, the quality control of mitochondria is essential to maintain cellular homeostasis and this process appears to be achieved via autophagy.

It has been postulated that mitophagy contributes to differentiation and development by participating to the intracellular remodelling that occurs for example during haematopoiesis and adipogenesis. In mammalian red blood cells, the expulsion of the nucleus followed by the removal of other organelles, such as mitochondria, are necessary differentiation steps. Nix/Bnip3L, an autophagy receptor whose structure resembles that of Atg32, is also an outer mitochondrial membrane protein that interacts with GABARAP [170, 171] and plays an important role in mitophagy during erythroid differentiation [172, 173] (Figure 3(c)). Although autophagosome formation probably still occurs in *Nix/Bnip3L* deficient reticulocytes, mitochondrial elimination is severely impaired. Consequently, mutant reticulocytes are exposed to increased levels of ROS and die, and *Nix/Bnip3L* knockout mice suffer severe anemia. Depolarization of the mitochondrial membrane potential of mutant reticulocytes by treatment with an uncoupling agent results in restoration of mitophagy [172], emphasizing the importance of Nix/Binp3L for the mitochondrial depolarization and implying that mitophagy targets uncoupled mitochondria. Haematopoietic-specific *Atg7* knockout mice also exhibited severe anaemia as well as lymphopenia, and the mutant erythrocytes markedly accumulated degenerated mitochondria but not other organelles [174]. The mitochondrial content is regulated during the development of the T cells as well; that is, the high mitochondrial content in thymocytes is shifted to a low mitochondrial content in mature T cells. *Atg5* or *Atg7* deleted T cells fail to reduce their mitochondrial content resulting in increased ROS production as well as an imbalance in pro- and antiapoptotic protein expression [175–177]. All together, these evidences demonstrate the essential role of mitophagy in haematopoiesis.

Recent studies have described the molecular mechanism by which damaged mitochondria are selectively targeted for autophagy, and have suggested that the defect is implicated in the familial Parkinson's disease (PD) [178] (Figure 3(c)). PINK1, a mitochondrial kinase, and Parkin, an E3 ubiquitin ligase, have been genetically linked to both PD and a pathway that prevents progressive mitochondrial damage and dysfunction. When mitochondria are damaged and depolarized, PINK1 becomes stabilized and recruits Parkin to the damaged mitochondria [122, 179–181]. Various mitochondrial outer membrane proteins are ubiquitinated by Parkin and mitophagy is then induced. Of note, PD-related mutations in *PINK1* and *Parkin* impair mitophagy [122, 179–181], suggesting that there is a link between defective mitophagy and PD. How these ubiquitinated mitochondria are recognized by the autophagosome remains unknown. Although p62 has been implicated in the recognition of ubiquitinated mitochondria, elimination of the mitochondria occurs normally in *p62*-deficient cells [182, 183].

### 4.5. Elimination of Invading Microbes

When specific bacteria invade host cells through endocytosis/phagocytosis, a selective type of autophagy termed xenophagy, engulfs them to restrict their growth [184] (Figure 3(d)). Although neither the target proteins nor the E3 ligases have yet been identified, invading bacteria such as *Salmonella enterica*, *Listeria monocytogenes,* or *Shigella flexneri* become positive for ubiquitin when they access the cytosol by rupturing the endosome/phagosome limiting membrane [185, 186]. These findings raise the possibility that ubiquitin also serves as a tag during xenophagy. In fact, to date, three proteins, p62 [105, 185, 187], NDP52 [188], and optineurin [115] have been proposed to be autophagy receptors linking ubiquitinated bacteria and LC3. An ubiquitin-independent mechanism has recently been revealed; recognition of a *Shigella* mutant that lacks the icsB gene requires the tectonin domain-containing protein 1 (Tecpr1), which appears to be a new type of autophagy adaptor targeting *Shigella* to Atg5- and WIPI-2-positive membranes [189]. Interestingly, the *Shigella* icsB normally prevents autophagic sequestration of this bacterium by inhibiting the interaction of *Shigella *VirG with Atg5 indicating that some bacteria have developed mechanism to inhibit or subvert autophagy to their advantage [190]. This latter category of pathogens also includes viruses such as Herpes simplex virus-1 (HSV-1), which express an inhibitor (ICP34.5) of Atg6/Beclin1 [106]. However, it was recently shown that a mutant HSV-1 strain lacking ICP34.5 becomes degraded by selective autophagy in a SMURF1-dependent manner [79], suggesting that selective autophagy plays an important role in our immune system.

Recently, a different antimicrobial function has been assigned to autophagy and this function appears to be selective. During infection, ribosomal protein precursors are transported by autophagy in a p62-dependent manner into lysosomes [112]. These ribosomal protein precursors are subsequently processed by lysosomal protease into small antimicrobial peptides. Importantly, it has been shown that induction of autophagy during a *Mycobacterium tuberculosis* infection leads to the fusion between phagosomes containing this bacterium and autophagosomes, and the production of the antimicrobial peptides in this compartment kills *M. tuberculosis* [112].

### 4.6. Lipophagy

While the molecular mechanism is largely unknown, autophagy contributes at least partially to the supply of free fatty acids in response to fasting (Figure 3(e)). Fasting provokes the increase of the levels of free fatty acids circulating in the blood, which are mobilized from adipose tissues. These free fatty acids are rapidly captured by various organs including hepatocytes and then transformed into triglycerides by esterification within lipid droplets. These lipid droplets appear to be turned over by a selective type of autophagy that has been named lipophagy in order to provide endogenous free fatty acids for energy production through *β*-oxidation [148]. Indeed, liver-specific *Atg7* deficient mice display massive accumulation of triglycerides and cholesterol in the form of lipid droplets [191]. Agouti-related peptide- (AgRP-) expressing neurons also respond to increased circulating levels of free fatty acids after fasting and then induce autophagy to degrade the lipid droplets [192]. Similar to the case in hepatocytes, autophagy in the neurons supplies endogenous free fatty acids for energy production and seems to be necessary for gene expression of AgPR, which is a neuropeptide that increases appetite and decreases metabolism and energy expenditure [192].

## 5. Conclusions and Perspectives

 Originally, it was assumed that autophagy was exclusively a bulk process. Recent experimental evidences have demonstrated that through the use of autophagy receptors and adaptors, this pathway can be selective by exclusively degrading specific cellular constituents. The list of physiological and pathological situations where autophagy is selective is constantly growing and this fact challenges the earliest concept whether autophagy can be nonselective. It is believe that under starvation, cytoplasmic structures are randomly engulfed by autophagosomes and delivered into the lysosome to be degraded and thus generate an internal pool of nutrients. In yeast *Saccharomyces cerevisiae*, however, the degradation of ribosomes, for example, ribophagy, as well as mitophagy and pexophagy, and the transport of the prApe1 oligomer into the vacuole under the same conditions requires the presence of autophagy receptors [97, 193–195]. As a result, these observations suggest that autophagy could potentially always operate selectively. This is a conceivable hypothesis because this process allows the cell to survive stress conditions and the casual elimination of cytoplasmic structure in the same scenario could lead to the lethal depletion of an organelle crucial for cell survival. Future studies will certainly provide more molecular insights into the regulation and mechanism of the selective types of autophagy, and this information will also be important to determine if indeed bulk autophagy exists.

## Abbreviations

AgRP: Agouti-related peptide
AMPK: AMP-activated protein kinase
ALFY: Autophagy-linked FYVE protein
Ams1: *α*-mannosidase 1
Ape1: Aminopeptidase 1
Ape4: Aminopeptidase 4
Atg: Autophagy-related gene
Bnip3L: B-cell leukemia/lymphoma 2 (BCL-2)/adenovirus E1B interacting protein 3
CK2: Casein kinase 2
CMA: Chaperone-mediated autophagy
Cvt: Cytoplasm to vacuole targeting
ER: Endoplasmic reticulum
FIP200: Focal adhesion kinase family interacting protein of 200 kD
FYCO1: FYVE and coiled-coil domain-containing protein 1
GABARAP: Gamma-aminobutyrate receptor-associated protein
GATE-16: Golgi-associated ATPase enhancer of 16 kDa
HDAC6: Histone de-acetylase 6
HOPS: Homotypic fusion and protein sorting
HSV-1: Herpes simplex virus-1
Keap1: Kelch-like ECH-associated protein 1
LC3: Microtubule-associated protein 1 (MAP1)-light chain 3
LIR: LC3-interacting region
LRS: LC3 recognition sequences
NBR1: Neighbour of BRCA1 gene
NDP52: Nuclear dot protein (NDP) 52
NF-*κ*B: Nuclear factor *κ*B
NIX: Nip-like protein X
Nrf2: NF-E2 related factor 2
PAS: Phagophore assembly site
PB1: Phox and Bem1p
PE: Phosphatidylethanolamine
PD: Parkinson's disease
PI3K: Phosphatidylinositol 3-kinase
PI3P: Phosphatidylinositol 3-phosphate
PKA: Protein kinase A
PKC: Protein kinase C
ROS: Reactive oxygen species
Rubicon: RUN domain and cysteine-rich domain containing Beclin 1-interacting protein
SMURF1: SMAD-specific E3 ubiquitin protein ligase 1
SUMO: Small ubiquitin-like modifier
SQSTM1: p62/sequestosome 1
TBK1: TANK binding kinase 1
Tecpr1: Tectonin domain-containing protein 1
TRAF6: Tumour necrosis factor receptor-associated factor 6
TOR: Target of Rapamycin
TGN: *Trans*-Golgi network
UBA: Ubiquitin associated
ULK1: Unc-51-like kinase 1
UVRAG: UV-resistance associated gen
Vps: Vacuolar protein sorting.
